# Quercetin-3-O-β-D-Glucopyranoside-Rich Fraction from *Spondias mombin* Leaves Halted Responses from Oxidative Stress, Neuroinflammation, Apoptosis, and Lipid Peroxidation in the Brain of Dichlorvos-Treated Wistar Rats

**DOI:** 10.3390/toxics10080477

**Published:** 2022-08-16

**Authors:** Olalekan Bukunmi Ogunro, Akeem Oni Salawu, Saqer S. Alotaibi, Sarah M. Albogami, Gaber El-Saber Batiha, Michel De Waard

**Affiliations:** 1 Department of Biological Sciences, Faculty of Applied Sciences, KolaDaisi University, Ibadan 200213, Nigeria; 2 Department of Biochemistry, Faculty of Life Sciences, University of Ilorin, Ilorin 240222, Nigeria; 3 Department of Biotechnology, College of Science, Taif University, Taif 21944, Saudi Arabia; 4 Department of Pharmacology and Therapeutics, Faculty of Veterinary Medicine, Damanhour University, AlBeheira 22511, Egypt; 5 L’Institut Du Thorax, CNRS, INSERM, Université de Nantes, F-44000 Nantes, France; 6 LabEx Ion Channels, Science and Therapeutics, F-06560 Valbonne, France

**Keywords:** dichlorvos, *Spondias mombin*, brain science, neurotoxicity, inflammation, quercetin-3-O-β-D-glucopyranoside, cognitive defects, antioxidant status, locomotion impairment, neurodegenerative diseases

## Abstract

**Highlights:**

- Protective efficacy of Quercetin-3-O-β-D-glucopyranoside-rich fraction (Q3G-RF) from *Spondias mombin* role on Dichlorvos (DDVP) toxicity was studied.
- DDVP induced cognitive and locomotive impairment, free radicals, oxidative stress, neuroinflammation, and apoptosis in the brain of rats.
- Q3G-RF from *S. mombin* leaves prevented DDVP-induced cognitive and locomotion impairment in the rats.
- Q3G-RF from *S. mombin* leaves prevented DDVP-induced deficits in fluid intake and body weight of the rats.
- Q3G-RF from *S. mombin* leaves abated DDVP-induced inhibition of AChE, BuChE in the brain sections of rats.
- Q3G-RF from *S. mombin* leaves reversed elevated activities APEH, DPP-IV and POP in the brain sections of rats exposed to DDVP.
- Q3G-RF from *S. mombin* leaves revoked the DDVP-induced oxidative stress in the brain sections of rats.
- Q3G-RF from *S. mombin* leaves prevented DDVP-induced neuroinflammation in the brain sections of rats.
- Q3G-RF from *S. mombin* leaves reversed DDVP-induced apoptosis in the brain sections of rats.
- Q3G-RF from *S. mombin* leaves assuaged DDVP neural toxicity in the brain sections of rats via anti-oxidative and anti-inflammatory mechanisms.

**Abstarct:**

The study investigated the prophylactic efficacy of Quercetin-3-O-β-D-glucopyranoside-rich fraction (Q3G-RF) from *Spondias mombin* leaves on oxidative and neuronal damages in the brain sections of dichlorvos (DDVP)-treated female Wistar rats. Female Wistar rats assigned into 5 groups of 12 rats each were orally treated with appropriate regimens. Group 1 received sunflower oil. Group 2 received DDVP (8.8 mg/kg). Group 3 received Q3G-RF (100 mg/kg). Group 4 received DDVP + Q3G-RF (50 mg/kg). Group 5 received DDVP + Q3G-RF (100 mg/kg). Q3G-RF reversed DDVP-induced cognitive deficits in the rats and reversed rearing activity impairment; it protected against the following DDVP-induced activities: inhibition of cholinesterases (acetylcholinestere (AChE), butyrylcholinesterase (BuChE)), elevated marker-enzymes (acylpeptide hydrolase (APEH), dipeptidylpeptidase IV (DPP-IV), prolyl oligopeptidase (POP)), oxidative stress (total thiol, malondialdehyde, hydrogen peroxide (H_2_O_2_), reduced glutathione and the activities glutathione S-transferase, catalase, superoxide dismutase, and glutathione peroxidase), inflammation (myeloperoxidase (MPO), tumor necrosis factor-α (TNF-α), nitric oxide, interleukin-1 beta (IL-1β)), and apoptosis (caspase-3) in the hippocampus, striatum, and prefrontal cortex of the experimental rats exposed to DDVP (*p* < 0.05). Conclusively, the neuroprotective effects of Q3G-RF on DDVP-induced toxicity in the hippocampus, striatum, and prefrontal cortex in brain of rats suggests its potent antioxidant, prophylactic, and chemotherapeutic properties.

## 1. Introduction

Dichlorvos (2,3-dichlorovinyl dimethyl phosphate or DDVP), is a popular organophosphate (OP) with several domestic, industrial, and agricultural uses and applications in developing countries [1,2]. Dichlorvos is used commercially in the protection of livestock and domesticated animals from parasite invasion, and in the control of insects and pests, but leaves residues in foods [3,4]. The quality of life of individuals or animals exposed to DDVP via ingestion, inhalation or absorption through the skin or eye contact has become a very big issue of public health concern [5,6]. Mortality resulting from acute DDVP poisonings is associated with respiratory failure from inhibition of central (medullary) respiratory drive, unrestrained bronchial secretions, and bronchospasms as well as depolarizing barricade at the diaphragm and intercostals (neuromuscular junctions) [7,8]. Dichlorvos exhibits several harmful effects not limited to cardiotoxicity, hepatotoxicity, immune system toxicity, renal toxicity, and hematological toxicity [1,8,9,10,11]. Studies have reported the neurotoxicity of DDVP in laboratory rats, but there is a paucity of information in respect to oxidative stress, neuroinflammation, apoptosis, lipid peroxidation, mitochondria dysfunction or aggregation of pathological proteins, that are regarded as the etiologies of neurodegeneration.

Meanwhile, acute intoxication of DDVP is caused by inhibiting acetylcholinesterase and by consequence, accumulates acetylcholine in the nervous system portion consisting of the brain and spinal cord, neural and gland linkages, and synapses involving the neural and muscle tissues (such as the smooth, skeletal, and cardiac muscles) [2]. This accumulation of acetylcholine disrupts the usual functions of the brain because of induced hyperexcitation in the cholinergic muscarinic and nicotinic receptors [12,13]. Furthermore, two enzymatic mechanisms are adopted for the breakdown of DDVP. The first involve the glutathione-independent mechanism during which dichloroacetaldehyde and dimethyl phosphate are produced by the action of catalyzed by esterases (‘A’ type). The other is glutathione-dependent mechanism that produces S-methyl glutathione and desmethyl dichlorvos [2]. Glutathione S-Transferase (GST, an enzyme having multiple functions including metabolizing xenobiotic to protect the brain from the harmful effect of drugs) therefore takes part in the detoxification of DDVP by catalyzing its conjugation with reduced glutathione (GSH). Aside from inhibition of cholinesterases, DDVP, like other OPs, disrupt normal oxidative functions [1]. Therefore, Glutathione S-Transferases are an important oxidative stress regulator.

Oxidative stress is speculated as an important mechanism involved in DDVP toxicity by reactions with different cells and tissues of the brain via polyunsaturated fatty acids (PUFAS)-rich biomembranes leading to cellular damage through lipid peroxidation [14]. Oxidative stress sets in normally when there is exuberant generation of free radicals, with a corresponding diminution of antioxidant enzymes such as superoxide dismutase (SOD), glutathione S-transferase (GST), catalase, glutathione reductase (GR), and glutathione peroxidase (GPx) [15,16] that could have helped to fight reactive oxygen species (ROS) in the brain in a normal state of the body or bodily functions [17,18]. However, with the accumulation of ROS, the brain may be damaged following the reciprocal action of generated ROS with lipids, carbohydrates, proteins, and DNA constituents of the brain [19]. Compared with other tissues, the brain on its own part is sensitivity to lipid peroxidation and susceptible to oxidative damage because it has high unsaturated lipids in peroxidizable form but low in content of antioxidants (both enzymatic and non-enzymatic) [20,21,22].

However, other plausible mechanisms by which DDVP affect the brain, which could be related to responses from oxidative stress, neuroinflammation, apoptosis, and lipid peroxidation, are vital. For instance, DDVP intoxication in the brain of laboratory animals may be by expressing machineries of pro-inflammation and apoptosis beyond the limit [23,24]. Inhibitors of acylpeptide hydrolase, dipeptidylpeptidase IV, and prolyl oligopeptidase in the brain therefore maybe useful drug leads to modulate the cellular metabolism in the mitochondria in the treatment of neurodegenerative diseases. Meanwhile, oxidative and neuronal damage of tissues by toxicants such as DDVP could be restored by active plant compounds. Plants such as *Spondias mombin* are therefore usually explored for their beneficial role against oxidative and neuronal damage associated with neurodegenerative diseases.

The leaves from *Spondias mombin* (popularly called hog plum—English, *Ichikara*—Igbos in Eastern Nigeria, *Tsardarmasar*—Hausa in Northern Nigeria and *Iyeye*—Yoruba in Southwestern Nigeria), have several ethnopharmacological properties acclaimed in folk medicine across cultures [25]. Akinmoladun et al. [26] reported that both fractions of ethyl acetate and n-butanol from *S. mombin* leaves yielded some novel compounds. Quercetin-3-*O*-β-D-glucopyranoside and undec-1-ene, among other compounds, were characterized from *S. mombin* leaves following chromatographic separation process. This suggests that the plant holds a promising source of chemical agents of various prophylactic and chemotherapeutic advantages. Hence, the interest in quercetin-3-*O*-β-D-glucopyranoside as a potential therapeutic and chemopreventive agent that is safe, efficacious and with multiple targets under control.

The present research thus studied the therapeutic efficacies of Q3G-RF against oxidative and neuronal damage by DDVP in the brain sections (hippocampus, stratum and prefrontal cortex) of female Wistar rats. Here, we examined the restorative effect of Q3G-RF on activities of AChE, BuChE, APEH, DPP-IV, POP. The effect of Q3G-RF on cognitive and locomotion performance as well as novel object recognition test was also examined. This study also assessed the protective capabilities of Q3G-RF on oxidative stress markers (malondialdehyde (MDA), H_2_O_2_, and total thiol), inflammatory markers (TNF-α, MPO, nitric oxide (NO), IL-1β), apoptotic marker (caspase-3), antioxidant enzymes (catalase, SOD, GPx, and GST) as well as GSH level in the hippocampus, stratum and prefrontal cortex of the brain of rats exposed to DDVP in order to provide more scientific perceptiveness on the efficacy of Q3G-RF as a therapeutic agent that can be explored in drug formulation against neurological-related disorders.

## 2. Materials and Method

### 2.1. Chemicals, Reagents, and Drugs

Dichlorvos, acetylthiocholine iodide, 5,5′-dithiobis (2-nitrobenzoic acid) (DTNB), glutathione, epinephrine, thiobarbituric acid, acetythiocholine, butyrylthiocholine, 1-chloro-2,4-dinitrobenzene, 5′,5′-dithiobis-2-nitrobenzoic acid–DTNB, tetramethylbenzidine, acetyl-alanine-*p*-nitroanilide, Gly-Pro-*p*-nitroanilide dithiothreitol, trichloroacetic acid (TCA), sunflower seed oil, and Tris–HCl buffer were products of Sigma-Aldrich (St. Louis, MO, USA). Acetic acid was obtained from British Drug Houses Chemical Ltd., (Poole, England). Enzyme-linked immunosorbent assay (ELISA) kits for interleukin-1 beta (IL-1β) and tumor necrosis factor-α (TNF-α) were products of ABCAM Scientific Corporation (England, UK) while Caspase-3 ELISA kit was a product of Elabscience Biotechnology Company (Beijing, China). All other reagents were of standard laboratory grade.

### 2.2. Plant Samples

Freshly harvested leaves of *Spondias mombin* from a healthy tree at Isaba-Ekiti, Nigeria were authenticated at the Department of Plant Biology, University of Ilorin, Ilorin, Nigeria (and a voucher sample allotted the number ‘UILH/001/1147′ was deposited at the Herbarium Unit).

The collected leaves of the plant were rinsed of sand using tap water and then dried, at 40 °C, to a constant weight using a laboratory oven (Surgifriend Medicals England, Devon, UK). The dried leaves having an invariant weight were then pulverized by an electric blender. The ethanol extract of *S. mombin* stem was thereafter prepared as described in the work of Ogunro and Yakubu [25] but with slight modifications. This involved a 96 h extraction of the resultant tiny loose particles (1000 g) in 98% ethanol (3 L), at 25 °C, by shaking the mixture at intervals. This process was then repeated twice. The resulting solution was first separated through fresh cotton bed and later passed through a Whatman No.1 filters paper to obtain a filtrate, later concentrated at a reduced temperature and pressure in a rotary vacuum evaporator. The concentrated ethanol extract was thereafter subjected to fractionation by adopting the Kupchan partitioning method but with slight modification [27]. The ethanol extract was fractionated into soluble fractions of butanol, hexane, and ethyl acetate. Hexane (9.5), ethyl acetate (10.7) and butanol (6.9) were afforded from the evaporation of the solvents. Meanwhile, fractionation of the ethyl acetate extract over silica gel was further performed using the Vacuum Liquid Chromatography (VLC) and solvents with increasing polarity. The resulting fraction from VLC (960 mg) with 20% MeOH in ethyl acetate was firstly made to undergo Gel Filtration Chromatography (2.3 g) that yielded 28 fractions after washing out with a solvent of chloroform (CHCl_3_)-Methanol (MeOH) mixtures. Sub fractions 4–16 were then combined (450 mg). The final step of Column Chromatography by washing out with 20% MeOH in ethyl acetate mixture subsequently afforded the quercetin-3-*O*-β-D-glucopyranoside-rich fraction (Q3G-RF) used in this study.

### 2.3. Animal Handling

Female Wistar rats (172 ± 3.18 g of about 16 weeks old without any physical or behavioral sign of estrous) were acclimatized in plastic cages in a well-ventilated animal house. Average temperature of 29 °C and humidity of 43% as well as 12 h light and dark cycle photoperiod were maintained throughout the experimental period in the animal house. The animals were equally maintained on rat chaws (Vital feeds^®^ from Jos, Nigeria) and clean water *ad libitum*. The animals were managed following the guide stipulated in Care and Use of Laboratory Animals of the National Academy of Science, issued by the National Institute of Health (Bethesda, MD, USA). 

The study was approved by the Research Ethic Committee of Ajayi Crowther University, Oyo (FNS/ERC/2022/020B), 15 February 2022 and was performed in accordance with the guidelines on the care and the use of laboratory animals.

### 2.4. Experimental Procedure and Treatment Grouping of the Rats

The animals totaling 60 female Wistar rats were randomly assigned into 5 groups of 12 rats each. They were orally administered with the appropriate regimen for 7 days as follows:

Group I: Sunflower oil only (2 mL kg^−1^ body weight)—sham control;

Group II: Dichlorvos dissolved in sunflower oil (8.8 mg/kg body weight)—negative control;

Group III: Q3G-RF (100 mg/kg body weight) only in sunflower oil—positive control;

Group IV: Dichlorvos (8.8 mg/kg body weight) and Q3G-RF (50 mg/kg body weight) concomitantly;

Group V: Dichlorvos (8.8 mg/kg body weight) and Q3G-RF (100 mg/kg body weight) concomitantly.

Thirty minutes before the final treatment regimens, the rats were subjected to cognitive performance test in Y-maze platform, novel object recognition test, and locomotor activity test. All administrations were performed daily at the same point time of between 8:00 am and 9:00 am, while the weights of the animals were recorded pre and post treatment times. The doses of DDVP was based on previous work of Imam et al. [28]. Because the safety tolerance of DDVP oral administration is very low, a 7-day treatment period was chosen.

### 2.5. Weight Measurement of the Total Body Mass and Brain of Rats

The body weights of the rats were measured using a weighing balance after they have adapted to new location of the experiment. Body weights on the first day of the experiment was noted as the initial weight, while body weights for the last day of treatment was taken as their final weight. Changes in weight was calculated as the differences between the final weight and initial weight. Weight of the whole brain for each rat was also recorded after sacrificing on the 8th day, and the values obtained were used to compute the brain-to-final body weight ratio in the present study.

### 2.6. Measurement of Cognitive Performance

#### 2.6.1. Y-Maze Test (YMT)

The effect of Q3G-RF on impairment of cognition was assessed as an indicator of spatial memory dysfunction using the Y-maze test [29]. The female rats were placed in a Y-shaped maze with three similar arms (tagged A, B, C) to conduct the test. Each arm of the maze was separated symmetrically at 120°, measuring 50 × 10 × 20 cm. Rats were monitored to demonstrate alternation behavior when they entered all three arms (either of ABC, CAB, or BCA but not BAB) in regular succession without gaps. Ability of the rats to move to a different arm option apart from the one previously occupied, whilst obtaining the right sequence of the visitation arms, was considered spontaneous alternation performance. Percentage correct alternation performance (an index of spatial working memory) was computed using:

$$\frac{\mathrm{Total}\;\mathrm{number}\;\mathrm{of}\;\mathrm{alternations}\times 100}{\left(\mathrm{Total}\;\mathrm{entry}\;\mathrm{number}-2\right)}$$

When each test period was concluded, ethanol (70%) was used to clean the maze at successive sessions to remove any residual odor from the previous animal. 

#### 2.6.2. Novel Object Recognition Test (NORT)

The novel object recognition test was carried out in a wooden observation chamber (60 × 50 × 40 cm). The animal was initially made to adapt to the environment by placing it in the empty open field for 10 min to explore the chamber [30]. Exactly four hours following the conformation phase, two identical objects (A and B) with a red-marked pattern were situated symmetrically 8 cm from the walls of the chamber and separated 34 cm from each other. The rats were placed same distance apart at every point from both objects and accorded the chance to examine minutely the objects in the chamber for 5 min. Each rat was returned to the experimental cage after the training session. In subsequent tests carried out after a day, the red-marked pattern object B was substituted with an entirely new object C with red-greenish patterns. The rat was returned to the box again and accorded the chance to examine minutely both objects A and C for 5 min. The period spent to examine each object (A and C) was recorded. The object examination period was defined as the moments spent by each rat to align the nose directly to the object in less than 2 cm and/or the use of the nose and whiskers to touch the object.

Examination excluded the animal climbing and sitting on the object. The chamber and objects were disinfected using ethanol (70%) to get rid of olfactory cues after successive sessions. Measurement of the recognition of the familiar object (Object A), termed discrimination index (DI) was computed as follows:

$$\mathrm{DI}=\frac{\mathrm{Time}\;\mathrm{expended}\;\mathrm{for}\;\mathrm{minute}\;\mathrm{examination}\;\mathrm{object}\;A\times 100}{\mathrm{Time}\;\mathrm{expended}\;\mathrm{for}\;\mathrm{minute}\;\mathrm{examination}\;\mathrm{of}\;\mathrm{both}\;\mathrm{Objects}\;A\;\mathrm{and}\;C}$$

#### 2.6.3. Measurement of Locomotion

The effect of Q3G-RF on locomotion in the experimental rats was assessed in an open field activity cage (Ugo-Basile, Gemonio, Italy), based on the counts of horizontal and vertical beam breaks [31]. The sensors that counted the beam breaks (both horizontal and vertical) quantified at 5 min intervals was what the activity detection depended on. The experimental cage was wiped with ethanol (70%) after successive test to get rid of olfactory remains of preceding animals.

### 2.7. Animals’ Sacrifice, Blood Collection and Brain Sectioning

Twenty-four hours after the last dose of Q3G-RF and dichlorvos as well as the performance of behavioral tests, the animals were denied food and water overnight, and their body weight was recorded as earlier described. From each group consisting of 12 animals, 10 were sacrificed by cervical dislocation. The whole brain was excised with care, washed off in frigid potassium chloride solution (0.15%), blotted and weighed. The brains were thereafter sectioned according to the procedural method of Tucker [32], while the striatum, prefrontal cortex, and hippocampus tissues were separated. These tissues were thereafter homogenized individually in ice-cold 0.1 M potassium phosphate buffer (pH 7.4) and then centrifuged at 12,000 rpm for 10 min, at 4 °C. Assays relating to oxidative stress, apoptosis, inhibition of enzymes, antioxidant state, and inflammation markers were carried out using the resultant supernatants in the present study.

### 2.8. Assay for Cholinesterase, Neurodegeneration Marker Enzymes, Neuroinflammation, Apoptosis, and Antioxidant Parameters 

#### 2.8.1. Cholinesterase and Brain Marker Enzymes for Neurodegeneration

##### Acetylcholinesterase (AChE) Activity

Determination of acetylcholinesterase activity in the hippocampus, striatum, and prefrontal cortex was performed by adopting the method of Ellman et al. [33]. The reaction was performed in K_3_PO_4_ buffer (0.1 M, pH 7.4), 5,5′-dithiobis (2-nitrobenzoic acid) (1 mM), and the initiator, acetylthiocholine (0.8 mM). The absorbance was measured at 412 nm every 30 s for 2 min. AChE activity was estimated as μmol of acetylthiocholine hydrolyzed/minute/mg protein.

##### Butyrylcholinesterase (BuChE) Activity

Determination of butyrylthiocholinesterase activity in the hippocampus, striatum, and prefrontal cortex was performed by adopting the method of Ellman et al. [33] described earlier. The reaction was performed in K_3_PO_4_ buffer (0.1 M, pH 7.4), 5,5′-dithiobis (2-nitrobenzoic acid) (1 mM), and the initiator, butyrylthiocholine (0.8 mM). The reaction was proceeded for 2 min (at 30 s intervals), while the absorbance was read at 412 nm. BuChE activity was expressed as μmol of butyrylthiocholine hydrolyzed/minute/mg protein.

##### Acylpeptide Hydrolase Activity

A slight modification of the Richards et al. [34] method was adopted for the assay of the activity of acylpeptide hydrolase (APEH) in the hippocampus, striatum, and prefrontal cortex. The assay mixture contained 4 mM acetyl-alanine-*p*-nitroanilide (AANA) and 0.1 M bis-Tris (pH 7.4). The absorbance (at 37 °C) was read at 405 nm. The enzyme activity was estimated as μmol of *p*-nitroaniline hydrolyzed/minute/mg protein measured as rate of appearance of *p*-nitroaniline.

##### Dipeptidylpeptidase IV Activity

For determination of the activity of dipeptidyl peptidase IV (DPP-IV) in the present study, the reaction mixture at 37 °C contained the supernatant (30 µL), 0.5 mM Gly-Pro-*p*-nitroanilide in 50 mM Tris.HCl of pH 7.4 (1 mL), and 1 mM dithiothreitol (DTT). The absorbance (at 37 °C) was read at 405 nm. The enzyme activity, measured as rate of appearance of *p*-nitroaniline, was estimated as μmol of *p*-nitroaniline hydrolyzed/minute/mg protein [34].

##### Prolyl Oligopeptidase Activity

Determination of activity of prolyl oligopeptidase enzyme in the hippocampus, striatum, and prefrontal cortex was performed according to the method of Cahlíková et al. [35]. The reaction mixture contained 30 µL of the supernatant, 0.25 mM *Z*-Gly-Pro-7-amino-4-methylcoumarin, 50 mM Tris.HCl, pH 7.4, and 1 mM DTT. The absorbance of the mixture was read with a spectrophotometer 383 nm (excitation wavelength) and 455 nm (emission wavelength), at 37 °C. The enzyme activity of POP was estimated as μmol of 7-amino-4-methylcoumarin released/minute/mg protein.

#### 2.8.2. Antioxidant Status Marker Enzymes

##### Glutathione S-Transferase Activity

Glutathione S-Transferase activity in the hippocampus, striatum, and prefrontal cortex was determined using the procedural steps in the method of Habig and Jakoby [36]. The substrate was 1-chloro-2,4-dinitrobenzene. Exactly 270 μL of a solution of potassium phosphate buffer (20 μL, 0.25 M pH 7.0) with EDTA (2.5 mM), distilled water (10.5 μL) and GSH (500 μL, 0.1 M) was mixed at 25 °C with CDNB (10 μL, 25 mM), and the sample (20 μL) made up the mixture. This reaction mixture was allowed to proceed for 5 min at time intervals of 10 s, and absorbance was read at 340 nm by a means of a spectrophotometer.

##### Glutathione Peroxidase Activity

The method of Rotruck et al. [37] was adopted in determining the activity of glutathione peroxidase in the hippocampus, striatum, and prefrontal cortex in this study. The reaction, which was stopped by 10% TCA (0.5 mL), contained mixture of sodium phosphate buffer (500 µL), 10 mM of sodium azide (100 µL), 4 mM GSH (200 µL), 2.5 mM H_2_O_2_ (100 µL), 50 µL sample made up to 2.0 mL with distilled water, 0.3 M disodium hydrogen phosphate (4 mL), and DTNB reagent (1 mL), incubated for 3 min, at 37 °C. The absorbance was read at a wavelength of 412 nm using a spectrophotometer, while the activity of glutathione peroxidase was expressed as units/mg protein. 

##### Catalase Activity

The catalase activity in the hippocampus, striatum, and prefrontal cortex was determined using the protocol of Aebi [38] in the present study. The mixture for the reaction consisted of 1800 µL of 50 mM potassium phosphate buffer (pH 7.0), 180 µL of 300 mM H_2_O_2_ and 20 µL of sample (1:50 dilution). The gradual decline in absorbance of H_2_O_2_, which was followed for 2 min at 240 nm, was utilized to evaluate the activity of catalase expressed as μmol of H_2_O_2_ depleted/minutes/mg protein.

##### Glutathione (GSH) Level

Glutathione level in the hippocampus, striatum, and prefrontal cortex was estimated using the method of Jollow et al. [39] in this study. An aliquot each of the samples was deproteinized by adding equal volume of 4% sulfosalicylic acid and then centrifuged at 10,000 rpm for 15 min, at 4 °C. This was followed by the addition of the 50 µL of the supernatants to obtain DTNB (10 mM, 4.5 mL). The absorbance was read at a wavelength of 412 nm, while the values were expressed in µmol/mg protein.

##### Superoxide Dismutase Activity

The Misra and Fridovich [40] method was employed in the determination of activity of superoxide dismutase (SOD). This was grounded on the ability to inhibit epinephrine autoxidation (pH 10.2) at 30 °C. Mixture for the reaction was 0.05 M carbonate–bicarbonate buffer (2.5 mL, pH 10.2) and the sample (20 µL). Adrenaline (0.3 mL, 0.3 mM) was then added and thoroughly mixed. Absorbance value at 480 nm in a spectrophotometer was monitored for 150 s at 30 s intervals for increments. The values were expressed as units of SOD activity/mg protein.

#### 2.8.3. Oxidative Stress Markers

##### Hydrogen Peroxide Generation

Hydrogen peroxide level in the hippocampus, striatum and prefrontal cortex was estimated by replicating the Wolff [41] method in this study. An amount of 100 mM xylenol orange (10 mL), (NH_4_)_2_Fe(SO_4_)_2_∙6H_2_O (50 mL, 250 mM), 100 mM sorbitol (10 mL), 25 mM H_2_SO_4_ (5 mL) and distilled water (30 mL) constituted the reaction mixture regarded as FOX 1, further combined with the homogenate sample. Incubation for 30 min at room temperature terminated the reaction, while the absorbance was read at 560 nm. The measures were derived through a known standard curve, while the results were elucidated in µmol/mg protein.

##### Total Thiol Content

The content of total thiol was estimated in accordance to the protocol reported by Ellman [42]. Exactly 20 μL of sample homogenate, 510 μL phosphate buffer (pH 7.4, 0.1 M), 35 μL DTNB (1 mM), and 35 μL distilled water constituted the reaction mixture. The reaction was terminated by incubating for 30 min, at room temperature. The absorbance was later read at 412 nm.

##### Malondialdehyde Level Determination

The Buege and Aust [43] method was adopted to quantify the level of malondialdehyde in the hippocampus, striatum, and prefrontal cortex in the present study. The reaction mixture contained 0.4 mL of the homogenate, 1.6 mL of Tris-KCl buffer, 0.5 mL of TCA (30%), 0.5 mL of TBA (0.75%). The absorbance was read at a wavelength of 532 nm in a spectrophotometer. The MDA formed representing the lipid peroxidation status was calculated considering the molar extinction coefficient of 1.56 × 105 m^−1^cm^−1^ estimated as µmol MDA mg^−1^ protein using the expression: Concentration of MDA (µmol MDA mg^−1^ protein) = (A532 − A600)/155.

#### 2.8.4. Neuroinflammation Markers

##### Nitric Oxide Level

Nitric oxide level in the hippocampus, striatum and prefrontal cortex was estimated by adopting the method of Griess reaction [44]. The procedural steps involved incubating the sample (250 μL) at room temperature with Griess reagent (250 μL) for 20 min. Absorbance was thereafter read at 550 nm using a spectrophotometer. Concentration of nitrite was estimated by comparing with the OD 550 of that of a standard solution of already established sodium nitrite concentrations.

##### Interleukin-1 Beta and Tumor Necrosis Factor-α Determination

Tumor necrosis factor-α (TNF-α) and interleukin-1 beta (IL-1β) levels were estimated in the hippocampus, striatum, and prefrontal cortex using the ELISA kits by strictly following the manufacturer’s guide.

##### Myeloperoxidase Activity Determination

Granell et al. [45] method was used to determine the activity of myeloperoxidase in the hippocampus, striatum, and prefrontal cortex in the present study. The homogenate supernatant (20 mL), 1.6 mM tetramethylbenzidine dissolved in dimethyl sulfoxide (10 mL), and 3 mM H_2_O_2_ (70 mL) diluted in 80 mM phosphate buffer (pH 5.4) constituted the reaction mixture. The absorbance was read at 630 nm using a spectrophotometer, while the activity of MPO was expressed as μM H_2_O_2_/min/mg protein.

#### 2.8.5. Apoptosis Marker

##### Caspase-3 Activity Determination

Caspase-3 activity was determined in the hippocampus, striatum and prefrontal cortex using the procured Caspase-3 ELISA kit by strictly following the procedural steps specified by the manufacturer. A microplate reader was employed to take readings of the absorbance at a wavelength of 450 nm, while the results were expressed as units/mg protein.

#### 2.8.6. Protein Concentration

The concentration of protein was estimated by the method of Lowry et al. [46] but with minor modifications.

#### 2.8.7. Statistical Analysis of Data

Data were presented as the mean ± SEM of 10 determinations. Determination of the significant differences among multiple groups in the respective treatments were performed using the one-way analysis of variance (ANOVA), proceeded by Newman-Keuls *post hoc* run. In all the groups, differences were taken to be significant among groups with *p* < 0.05, with the use of Prism^®^ ver. 5.01 (GraphPad Software, Inc., La Jolla, CA 92037, USA).

## 3. Results

### 3.1. Q3G-RF from S. mombin Leaves Reversed DDVP-Induced Cognitive and Memory Impairment

The impact of Q3G-RF on cognitive performance of rats treated with DDVP is depicted in Table 1. Q3G-RF reversed DDVP-induced cognitive deficits in rats by increasing the percent of correct alternation in the Y-maze paradigm and improved cognitive performance evidenced as an increase in DI using the novel object recognition paradigm.

### 3.2. Q3G-RF from S. mombin Leaves Improved DDVP-Induced Locomotion Impairment

The efficacy of Q3G-RF against locomotion impairment caused by DDVP is shown in Table 1. DDVP caused a reduction in spontaneous motor activity as indicated by the horizontal beam breaks counts in 5 min, but treatment with Q3G-RF in the doses (50 and 100 mg/kg body weight) increased horizontal beam breaks and reversed rearing activity impairment/exploratory behavior, which prevented the rats from moving around the environment. Co-treatment of DDVP with the 100 mg/kg body weight of Q3G-RF demonstrated a better effect when compared to the sham control.

### 3.3. Q3G-RF from S. mombin Leaves Prevented DDVP-Induced Body Weight Deficit in Rats

**Figure 1:**
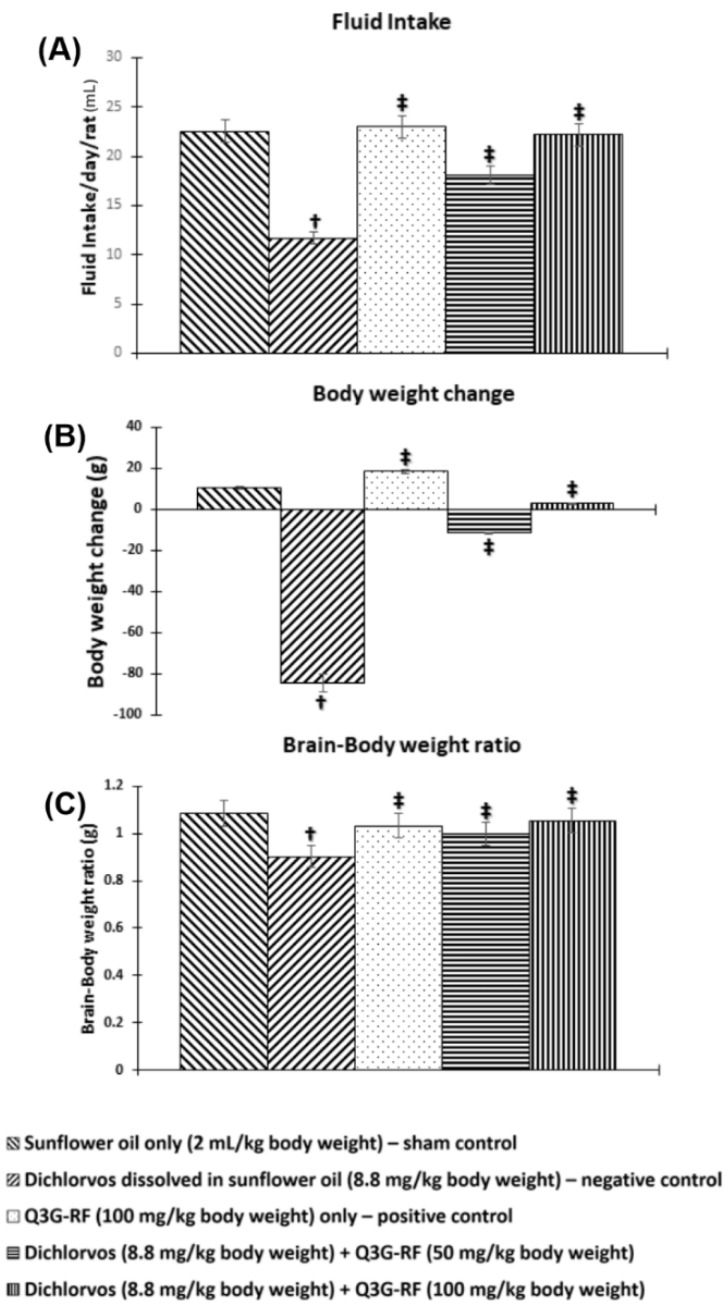
Influence of Q3G-RF from *S. mombin* leaves on feeding and anthropometric indices. Fluid intake (**A**), body weight difference (**B**) and brain/body weight ratio (**C**) of Wistar rats treated with sunflower oil, DDVP alone, Q3G-RF alone, and DDVP + Q3G-RF (50 and 100 mg/kg). Values are expressed as mean ± standard error of mean (*n* = 10). Significant differences from the positive control group are indicated by **†** and from DDVP alone by **‡**, (*p* < 0.05). DDVP, Dichlorvos; Q3G-RF, Quercetin-3-O-β-D-glucopyranoside-rich fraction from *Spondias mombin*.

The effect of Q3G-RF and DDVP on body weight difference in rats is depicted in Figure 1. When compared with the control, DDVP alone significantly (*p* < 0.05) reduced the fluid intake (Figure 1A), body weight (Figure 1B), and brain–body weight ratio (Figure 1C) of the experimental rats. However, co-treatment of DDVP with Q3G-RF significantly improved the fluid intake, body weight, and brain–body weight ratio compared with the DDVP-alone-treated rats (*p* < 0.05). Co-treatment of DDVP with the highest dose of Q3G-RF (100 mg/kg body weight) compared favorably with the control. Furthermore, Q3G-RF alone significantly increased the value of fluid intake, body weight, and brain–body weight ratio when compared to the control group treated with sunflower oil only (*p* < 0.05).

### 3.4. Q3G-RF from S. mombin Leaves Reversed the Inhibition of Cholinesterases and Alteration in Activities of Brain Enzymes of Rats Exposed to DDVP

**Figure 2:**
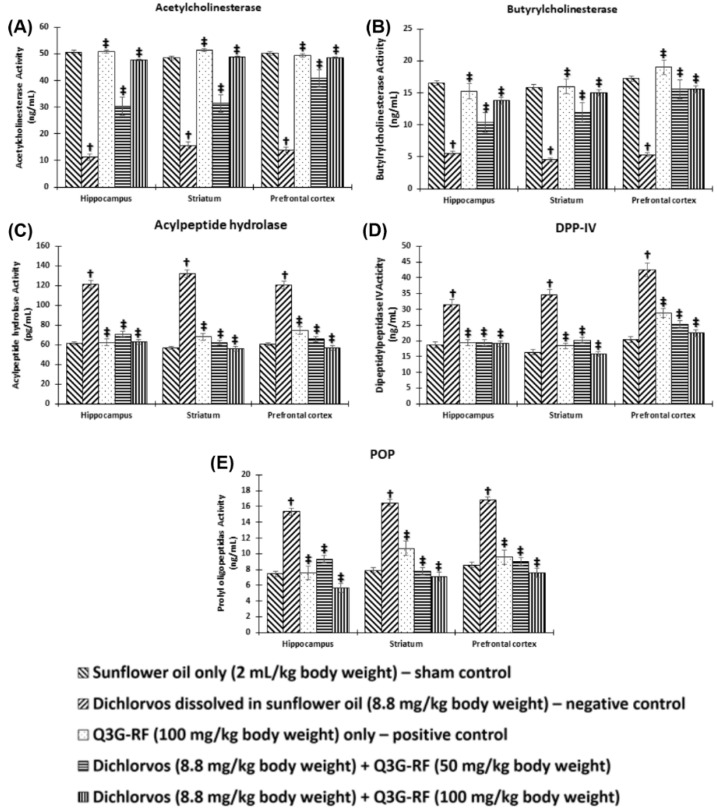
Capacity of Q3G-RF from *S. mombin* leaves to reverse the inhibition of cholinesterases and alteration in activities of brain enzymes exposed to DDVP. Activities of AChE (**A**), BuChE (**B**), APEH (**C**), DPP-IV (**D**) and POP (**E**) of Wistar rats treated with sunflower oil, DDVP alone, Q3G-RF alone, and DDVP + Q3G-RF (50 and 100 mg/kg). Values are expressed as mean ± standard error of mean (*n* = 10). Significant differences from the positive control group are indicated by **†** and from DDVP alone by **‡**, (*p* < 0.05). DDVP, Dichlorvos; Q3G-RF, Quercetin-3-O-β-D-glucopyranoside-rich fraction from *Spondias mombin*.

The effect of Q3G-RF on the brain activities of AChE, BuChE, APEH, DPP-IV and POP in the hippocampus, striatum, and prefrontal cortex of rats treated with DDVP is shown in Figure 2. DDVP alone significantly (*p* < 0.05) inhibited the activities of AChE Figure 2A), BuChE (Figure 2B) but significantly elevated the activities of APEH (Figure 2C), DPP-IV (Figure 2D), and POP (Figure 2E) in hippocampus, striatum, and prefrontal cortex tissues when compared with control rats (Figure 2). 

However, co-administration of DDVP with Q3G-RF (either at 50 and 100 mg/kg body weight) attenuated the activities of AChE, BuchE, APEH, DPP-IV, and POP in the hippocampus, striatum, and prefrontal cortex tissues in a dose-related manner when compared with the DDVP-alone-treated rats (*p* < 0.05). Furthermore, in rats treated with only Q3G-RF, the activities of AChE and BuchE were increased, while the activities of APEH, DPP-IV, and POP were reduced when compared with the sham control treated with sunflower oil only (*p* < 0.05).

### 3.5. Q3G-RF from S. mombin Leaves Improved Antioxidant Status in Rats Exposed to DDVP

**Figure 3:**
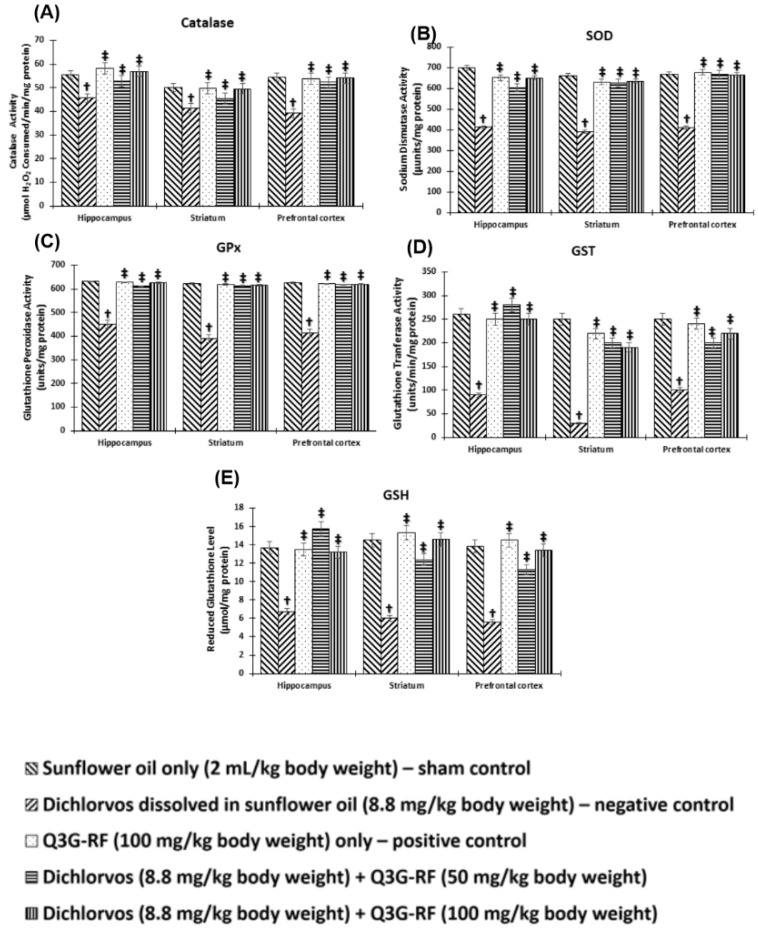
Efficacy of Q3G-RF from *S. mombin* leaves on antioxidant status in the brain of DDVP-induced neural impairment of female Wistar rats. Activities of Catalase (**A**), SOD (**B**), GPx (**C**), and GST (**D**) as well as GSH level (**E**) of Wistar rats treated with sunflower oil, DDVP alone, Q3G-RF alone, and DDVP + Q3G-RF (50 and 100 mg/kg). Values are expressed as mean ± standard error of mean (*n* = 10). Significant differences from the positive control group are indicated by **†** and from DDVP alone by **‡**, (*p* < 0.05). DDVP, Dichlorvos; Q3G-RF, Quercetin-3-O-β-D-glucopyranoside-rich fraction from *Spondias mombin*.

The 7-day exposure of rats to DDVP and Q3G-RF on selected antioxidant parameters in the hippocampus, striatum, and prefrontal cortex of rats is shown in Figure 3. Relative to the control, DDVP alone, significantly (*p* < 0.05) reduced the activities of catalase (Figure 3A), SOD (Figure 3B), GPx (Figure 3C), and GST (Figure 3D) and level of GSH (Figure 3E). In contrast, co-treatment of DDVP with Q3G-RF at either 50 or 100 mg/kg body weight significantly (*p* < 0.05) increased the activities of catalase, SOD, GPx, GST and the level of GSH when compared with rats treated with only DDVP. The effect of Q3G-RF when co-administered with DDVP was dose-dependent, and the highest dose compared favorably well with the control. Moreover, rats treated with only Q3G-RF also had significant increases in activities of catalase, SOD, GPx, GST and the level of GSH compared to the control when compared to the sham control-administered sunflower oil only (*p* < 0.05).

### 3.6. Q3G-RF from S. mombin Leaves Prevented DDVP-Induced Oxidative Stress

**Figure 4:**
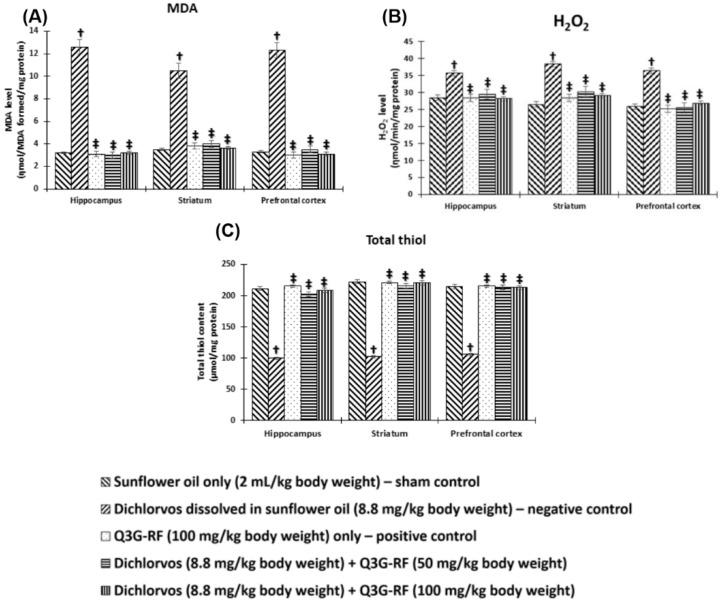
Impact of Q3G-RF from *S. mombin* leaves on oxidative stress markers in the brain of DDVP-induced neural impairment of female Wistar rats. MDA (**A**), H_2_O_2_ (**B**), and total thiol content (**C**) of Wistar rats treated with sunflower oil, DDVP alone, Q3G-RF alone, and DDVP + Q3G-RF (50 and 100 mg/kg). Values are expressed as mean ± standard error of mean (*n* = 10). Significant differences from the positive control group are indicated by **†** and from DDVP alone by **‡**, (*p* < 0.05). DDVP, Dichlorvos; Q3G-RF, Quercetin-3-O-β-D-glucopyranoside-rich fraction from *Spondias mombin*.

The effect of Q3G-RF on brain H_2_O_2_ and MDA levels, as well as total thiol content in the hippocampus, striatum, and prefrontal cortex of rats treated with DDVP is depicted in Figure 4. When compared to the control group, DDVP alone, significantly (*p* < 0.05) increased the levels of MDA (Figure 4A) and hydrogen peroxide (Figure 4B) but reduced the total thiol content (Figure 4C). However, co-exposure of DDVP with Q3G-RF at 50 and 100 mg/kg body weight abrogated the increase in MDA and H_2_O_2_ level while the thiol content was increased when compared with the DDVP-alone-treated group (*p* < 0.05). Co-exposure of DDVP with 100 mg/kg body of Q3G-RF demonstrated a better effect when compared to the control. In addition, Q3G-RF alone, significantly attenuated the levels of MDA and H_2_O_2_ and correspondingly increased the total thiol content when compared with the show control-administered sunflower oil only (*p* < 0.05).

### 3.7. Q3G-RF from S. mombin Leaves Prevented DDVP-Induced Neuroinflammation and Apoptosis

**Figure 5:**
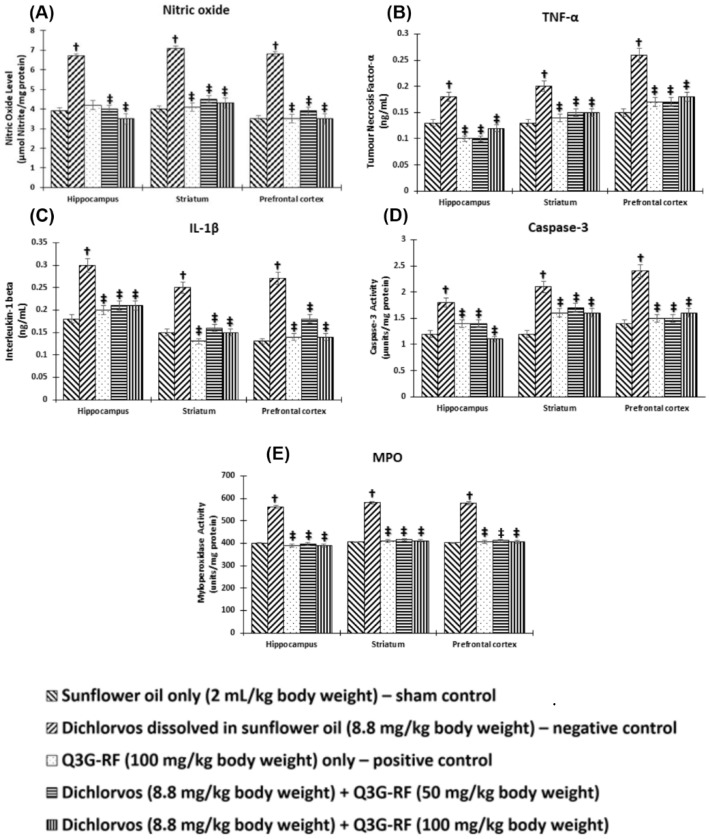
Effects of Q3G-RF from *S. mombin* leaves on inflammation and apoptotic markers in the brain of DDVP-induced neural impairment of female Wistar rats. NO (**A**), TNF-α (**B**), IL-1β (**C**), as well as activities of caspase 3 (**D**), and MPO (**E**) of Wistar rats treated with sunflower oil, DDVP alone, Q3G-RF alone, and DDVP + Q3G-RF (50 and 100 mg/kg). Values are expressed as mean ± standard error of mean (*n* = 10). Significant differences from the positive control group are indicated by **†** and from DDVP alone by **‡**, (*p* < 0.05). DDVP, Dichlorvos; Q3G-RF, Quercetin-3-O-β-D-glucopyranoside-rich fraction from *Spondias mombin*.

The effect of Q3G-RF on the brain activity of MPO and caspase 3 as well as levels of NO, TNF-α, and IL-1β in the hippocampus, striatum, and prefrontal cortex of rats treated with DDVP is shown in Figure 5. DDVP only significantly (*p* < 0.05) increased the levels of NO (Figure 5A), TNF-α (Figure 5B), IL-1β (Figure 5C) as well as the activities of caspase (Figure 5D) and MPO (Figure 5E) when compared to the control. 

However, co-treatment of DDVP with Q3G-RF at 50 and 100 mg/kg body weight attenuated the levels of NO, TNF-α, and IL-1β as well as the activities of MPO and caspase-3 in a dose-dependent manner when compared with the rats treated with DDVP alone. Furthermore, Q3G-RF alone significantly reduced the activities/levels of NO, TNF-α, IL-1β, MPO and caspase-3 compared to the sham control-administered sunflower oil only. 

## 4. Discussion

The brain contains several billions of nerve cells enabled to send and receive signals to other parts of the human body through the nervous system. Major organs and senses obtain information from the cells or neurons of the brain and thereby coordinate the vital processes of life. Any damage to the nerve cells therefore affects the quality of life both physically and mentally [47]. Additionally, damage to the nervous system may either be a slow process with gradual declination of the functions, or it could be sudden and life-threatening. While the former is degenerative, the latter is termed acute [48,49]. Whichever the case, a perfect understanding of the occurrence and the impact are germane for treating neurodegenerative diseases. 

The brain is highly vulnerable to DDVP-induced damage, due to its low content of protective enzymes. Speculation on the causes of this damage include increased production of free radicals/oxidative stress, apoptosis, and mitochondrial dysfunction, all conditions implicated in neurodegenerative disorders. Apoptosis is an active mode of cell death known as programmed cell death, in which diseased cells die. Oxidative stress sets in normally when there is exuberant generation of free radicals, with a corresponding diminution of antioxidant enzymes such as superoxide dismutase, glutathione S-transferase, catalase, glutathione reductase, and glutathione peroxidase [15,16] that could have helped to quench ROS in the brain in a normal state of the body [50,51]. However, with the accumulation of ROS, the brain may be damaged following the reciprocal action of generated ROS with lipids, carbohydrates, proteins, and DNA constituents of the brain [19]. Sadly, oxidative stress forms an important mechanism involved in DDVP toxicity by reactions with different cells and tissues of the brain via PUFAS-rich biomembranes, leading to cellular damage through lipid peroxidation [14]. DDVP as an organophosphate pesticide is widely distributed in environment for agricultural and household uses and had detrimental effect to the quality of life when ingested. It is therefore of significance to provide substantial evidence on the protective efficacy of Q3G-RF against the following DDVP-induced activities: inhibition of cholinesterases (acetylcholinesterase and butyrylcholinesterse), elevation of brain marker enzymes (acylpeptide hydrolase, dipeptidylpeptidase IV, prolyl oligopeptidase), oxidative stress (hydrogen peroxide, total thiol, and malondialdehyde), neuroinflammation (myeloperoxidase, nitric oxide, tumor necrosis factor-α, interleukin-1 beta), apoptosis (caspase-3), and disruption of antioxidant status (catalase, SOD, GPx, GST and GSH), in the hippocampus, striatum, and prefrontal cortex of rats. Additionally, the effect Q3G-RF on DDVP-induced loss in body weights, cognitive and locomotive impairment of rats after treatment for 7 days of exposure was reported. 

Animal body weights during in vivo studies are usually used as an index to assess the overall health conditions [52]. The significant loss of body weight following exposure to DDVP suggests distress and pain in the animals. This also corroborates the reduction in fluid intake. Previous studies have established a link between DDVP and loss of brain and body weight. This condition can also be associated with loss of biological functions in the cells, which may contribute to the pathophysiological factors for life-threatening diseases including neurodegenerative disorders [1,28,53]. The significant increase in fluid intake and weight gain effected by Q3G-RF suggest its potency to reverse the pathophysiological conditions detrimental to life and proper functioning of the cells and tissues. It also suggests the immunoregulatory effect of Q3G-RF being able to abrogate cytokine interleukin-6 (IL-6) in this study, in which elevation in the brain has been linked to the reduction in food intake and exacerbation of sickness behavior [54].

Moreover, development of healthy nerve tissues of a healthy hippocampus enhances psycho-cognitive functions, but these cognitive functions are affected when there is any damage that affects neurogenesis of the hippocampus [55]. Reduction in correct alternation behavior and the amount of time exploring novel objects in the NORT by animals exposed to DDVP in the present study indicates cognitive dysfunctions in psychosocial-related functions in the brain of the rats. Treatment with Q3G-RF increased the cognitive performance in rats which suggests its potency against conditions associated with memory loss or neurocognitive deficits [1,53]. This finding is in tandem with reports by Ishola et al. [56] and Ajayi et al. [57] which confirms the anti-amnesic potential of the leaves and fruits of the plant. It also suggests Q3G-RF as anti-inflammatory agent against cytokines such as IL-6, whose elevation could have inhibited memory and learning as well as neurodegeneration in this study.

Acetylcholinesterase (AChE) and butyrylcholinesterase (BuChE) are the two major cholinesterases in the brain of man. DDVP has been known to be inhibit the activities of acetylcholinesterase [58,59] with reports confirming the entry of DDVP into the brain. Acetylcholinesterase is involved in the termination of impulse transmission by rapid hydrolysis of the neurotransmitter acetylcholine in numerous cholinergic pathways of the central and peripheral nervous systems. Acetylcholinesterase inhibitors, interacting with the enzyme as their primary target, are applied as relevant drugs and toxins. The inhibition in the activities of acetylcholinesterase and butyrylcholinesterase in the hippocampus, striatum, and prefrontal cortex of rats by DDVP in the present study is an indication of accumulated acetylcholine probably from cholinergic degeneration [60]. This is in tandem with previous reports which suggest impairment of the memory and learning processes in the present study [28,61]. The countereffect of Q3G-RF on acetylcholinesterase and butyrylcholinesterase in the hippocampus, striatum, and prefrontal cortex of rats can be related to the ability to overturn responses from neuroinflammation, oxidative stress, apoptosis, and pathological proteins aggregation.

Additionally, acylpeptide hydrolase, dipeptidyl peptidase IV, and prolyl oligopeptidase inhibitors presumed to find relevance as drug lead in the cure of neurodegenerative diseases implied their assay in the present study. For instance, acylpeptide hydrolase is assumed to take part in the regulation of neuropeptide turnover, thus suggesting a new drug lead and plausible mechanism towards its effects in enhancing cognition and reducing neurodegeneration [62,63], while prolyl oligopeptidase (POP) is prominently expressed in the brain and has been implicated in neuroinflammatory events in neurodegenerative diseases. POP protein levels are increased in brain glial cells upon neuroinflammatory insults, while its inhibition enhances cognition and neuroprotective effects in animal models with cognitive deficits [64,65]. DPP-IV is upregulated in AD brain neurons and relegates the functions of several chemokines, cytokines, and neuropeptides that influences immunity, inflammation, and vascular function that are normally released in the blood stream through its enzymatic activity [66,67]. Increased activities of acylpeptide hydrolase, dipeptidyl peptidase, and prolyl oligopeptidase by DDVP suggest neuroinflammation of the hippocampus, striatum, and prefrontal cortex of the rats or alteration in the pathways of cellular metabolism in the mitochondria. In contrast, the ability of Q3G-RF to decrease the activities of these enzymes suggests its therapeutic efficacy and neuroprotective effects to abrogate cognitive loss resulting from Alzheimer’s disease and related dementias. The blockage in the expression of DPP-IV and APEH by Q3G-RF suggest its direct immunosuppressive efficacy therefore providing a bright insight towards therapeutic development of drugs for the treatment of neurological conditions of immune-related dysfunctions [63,68]. The inhibition of POP by Q3G-RF further substantiates its neuroprotective effects and efficacy in enhancing cognition in animal models with cognitive deficits.

As previously opined in this study, oxidative stress is an important mechanism involved in DDVP toxicity by reactions with different cells and tissues of the brain. Therefore, the DDVP-induced oxidative stress that resulted in the activities of catalase, SOD and GPx in the hippocampus, striatum, and prefrontal cortex of rats coupled with the increase in level of H_2_O_2_ in the present study is an indication of oxidative damage suggesting impairment of the antioxidant status. Ajiboye [69] and Sharma and Singh [70] reported a similar decrease in the activities of catalase and SOD in the brain of rats following DDVP intoxication, while Ojo et al. [14] and Ochigbo et al. [71] reported the decreased activity of GPx in the brain of rats exposed to DDVP. The restoration in the activities of catalase, SOD and GPx in the hippocampus, striatum, and prefrontal cortex of rats as well as the level of H_2_O_2_ suggest the chemoprotective potential of Q3G-RF by suppressing lipid peroxidation in the brain tissues.

Meanwhile, some studies have previously reported the decreased activity and level of GST and GSH respectively. The decrease in GST activity in the present study can be linked to the inhibition of the enzyme by the metabolites of DDVP as well as excessive availability of MDA, a lipid peroxidation product. This also aligns with the reduction in the level of GSH (a vital cofactor for antioxidant enzymes such as GST and GPx as well as a non-enzymatic antioxidant in tissues) in the present study because it functions to conjugate electrophilic metabolites of DDVP. Thus, the DDVP-induced reduction in GSH level implies oxidative stress in the hippocampus, striatum, and prefrontal cortex of the experimental rats. The reductions in the activity of GST and level of GSH by DDVP in the present study are similar to such reported by Ajiboye in the whole brain of experimental rats [69]. In contrast, the ability of Q3G-RF to restore the DDVP-induced inhibition of GST and GSH in the brain suggests its ability to mop up reactive oxygen species in the brain as an agent with neuroprotective potentials to eliminate toxicants.

Furthermore, lipid peroxidation is one of the molecular mechanisms by which several toxicants including DDVP exhibit their harmful effects especially in the brain. For instance, the sensitivity of the brain to lipid peroxidation is markedly high because of its free fatty acids’ copiousness [22]. The increase in the marker of lipid peroxidation, MDA levels in the present study by DDVP is an indication that the brain of the rats might be undergoing cellular damages and dysfunction from excess production of highly reactive oxygen free radicals such as hydroxyl radicals, which interact with unsaturated fatty acids of phospholipids components of the brain [57]. This corroborates the reduced glutathione level, which is regarded as the first indicator of oxidative stress in this study. A similar observation of marked increase in cerebellar MDA levels of experimental rats was previously reported by Ochigbo et al. [71]. However, the ability of Q3G-RF to normalize MDA levels comparable with the respective control may be linked to its antiperoxidative properties.

Hydrogen peroxide is the most measured ROS, due to its stability when compared with superoxide and hydroxyl radicals. It is harmful to the brain because it readily crosses their membranes to induce toxicity [72]. Myeloperoxidase on its own acts as a peroxidase by converting hydrogen peroxide and chloride ions to hypochlorous acid, thereby participating in tissue defense [73]. Therefore, the DDVP-induced increases in the level of H_2_O_2_ and the activity of MPO in the hippocampus, striatum, and prefrontal cortex of the experimental rats in this study indicate accumulation of hydrogen peroxide accrued from the toxicity of DDVP. The accumulated hydrogen peroxide could also be responsible for the functional increase in oxidative phosphorylation and oxidative stress. Ajiboye [69] and Ochigbo et al. [71], in separate studies, have reported related findings of the increased level of H_2_O_2_ but in the whole brain of the experimental rats. Meanwhile, the ability of Q3G-RF to modulate the level of H_2_O_2_ and MPO activity in the brain suggests its antioxidant efficacy against accumulation of ROS and oxidative stress response.

Neuroinflammation, among other defensive mechanisms is one of the ways by which the nervous system of a host is protected from neurotoxic assaults. Neuroinflammation is also one of the most common pathological outcomes or etiologies of neurodegeneration and neurological disorders, which makes it a promising target of clinical importance [74]. Hence, the essence of screening selected neuroinflammation markers in this study. For instance, tumor necrosis factor-α (TNF-α) being a pleiotropic mediator influences several physiological and neurological functions including responses to toxicants and the usual regulatory functions [75], while cytokine interleukin-6 (IL-6) plays a vital role in physiological homeostasis of neural tissue and in the pathogenesis of inflammatory disorders [76]. Nitric oxide, while serving a retrogressive neurotransmitter role in synapses, allows for the brain blood flow and plays a vital role in intracellular signaling in neurons [77]. Myeloperoxidase is a vital inflammatory enzyme and therapeutic target related to pathological processes that triggers oxidative stress and neuroinflammation [78]. The increase in the levels of TNF-α and IL-6 in the hippocampus, striatum, and prefrontal cortex suggest interference with the normal physiological homeostasis of the neural tissues linked to neuroinflammation, since the elevation of both TNF-α and IL-6 are associated with the pathogenesis of neurodegenerative diseases. DDVP might have also compromised intracellular signaling in the brain, which could be the reason for the elevated level of nitric oxide in the present study. The reduction in food intake and exacerbation of sickness behavior as well as inhibition of memory and learning in the present study can also be linked with elevated IL-6 [54]. The increase in the activity of MPO in the hippocampus, striatum, and prefrontal cortex can also be associated with the depleted antioxidant status and increased oxidative stress in this study. In contrast, the ability of Q3G-RF to restore the DDVP-induced elevation of NO and TNF-α confirms that it can serve as a potential anti-inflammatory agent in brain diseases mediated by inflammation.

**Scheme 1:**
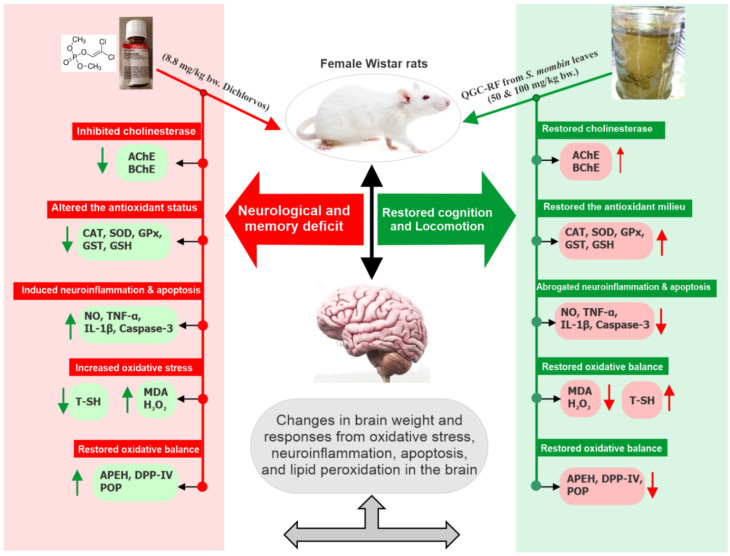
Neuroprotective, anti-apoptotic, anti-inflammatory, and anti-oxidative mechanism by which Q3G-RF from *S. mombin* leaves reversed neural toxicity of DDVP in the brain of Wistar rats.

Accumulative ROS and depletion of antioxidant enzymes exhibit a critical role in cell death and apoptosis. DDVP has been reported to induce apoptosis in neuronal cells because of mitochondrial dysfunction [23], because the mitochondrial membrane depolarization can cause release of cytochrome c followed by caspases activation and, therefore, activation of the intrinsic apoptotic pathway. The enhancement of the activity of caspase-3 (cysteine-dependent aspartate-directed protease-3) in the hippocampus, striatum, and prefrontal cortex of the experimental rats could be linked to pathological disease conditions in the brain. However, with Q3G-RF, the DDVP-induced apoptosis from the increase in caspase-3 activity was restored to normal, which suggests the antiapoptotic property necessary for boosting cell differentiation and immunity. The result from the present study suggests the possibility of Q3G-RF involvement of mitochondrial pathway in cell apoptosis caused by DDVP (Scheme 1).

In conclusion, DDVP induced oxidative damage, neuroinflammation, and apoptosis in the brain of rats. However, quercetin-3-O-β-D-glucopyranoside-rich fraction from *Spondias mombin* leaves offered protective effects on DDVP-induced toxicity in the striatum, prefrontal cortex, and hippocampus tissues, thus affirming its potent antioxidant, prophylactic, and chemotherapeutic properties against responses from oxidative stress, neuroinflammation, apoptosis, and lipid peroxidation in the brain of Wistar rats exposed to DDVP.

## Figures and Tables

**Table 1:** Impact of Q3G-RF from *S. mombin* leaves on cognitive performance and locomotion impairment in DDVP-treated female Wistar rats.

Treatment Dose/Grouping	Cognitive Deficits		Locomotor Impairment	
	Correct Alternations (%) ^a^	Discrimination Index ^a^	Spontaneous Motor Activity (Horizontal Beam Breaks/5 min) ^a^	Discrimination Index (Vertical Beam Breaks/5 min) ^a^
Control (2 mL of sunflower oil)	82.56 ± 2.11	77.34 ± 1.30	622.06 ± 10.30	50.77 ± 1.30
DDVP (8.8 mg/kg) + Sunflower oil	44.67 ± 1.12 ^†^	40.56 ± 0.30 ^†^	301.78 ± 10.23 ^†^	26.77 ± 1.22 ^†^
Q3G-RF (100 mg/kg)	93.78 ± 2.23 ^‡^	89.86 ± 2.10 ^‡^	631.98 ± 10.12 ^‡^	54.27 ± 2.30 ^‡^
DDVP (8.8 mg/kg) + Q3G-RF (50 mg/kg)	67.78 ± 1.14 ^‡‡^	60.78 ± 1.90 ^‡‡^	568.00 ± 11.60 ^‡^	43.33 ± 1.33 ^‡‡^
DDVP (8.8 mg/kg) + Q3G-RF (100 mg/kg)	78.89 ± 1.23 ^‡^	75.67 ± 1.99 ^‡^	599.98 ± 14.50 ^‡^	48.43 ± 1.80 ^‡^

^a^ Data are mean of 10 determinants ± SEM. ^†^ *p* < 0.05 vs. control; ^‡^ *p* < 0.05 vs. control, ^‡‡^ *p* < 0.01 vs. sunflower oil utilizing the one-way analysis of variance (ANOVA), proceeded by Newman-Keuls *post hoc* run. **DDVP**, Dichlorvos; **Q3G-RF**, Quercetin-3-O-β-D-glucopyranoside-rich fraction from *Spondias mombin*.

## Data Availability

The raw data can be tendered upon reasonable request.

## Abbreviations

AChE, acetylcholinesterase; BChE, butyrylcholinesterase; DOPA, dopamine; APEH, acyl peptide hydrolase; DPP-IV, dipeptidyl peptidase IV; POP, prolyl oligopeptidase, MDA, malondialdehyde; MPO, myeloperoxidase; TNF-α, tumor necrosis factor-α; IL-1β, interleukin-1; H_2_O_2_, Hydrogen peroxide; NO, Nitric oxide; CAT, catalase; GPx; Glutathione peroxidase,; ROS, Reactive oxygen species; GST, Glutathione S-Transferase; GSH, Reduced Glutathione; TBARS, Thiobarbituric acid reactive substances; T-SH, Total thiol; HO·, Hydroxyl radical; DTNB, 5,5′-dithiobis (2-nitrobenzoic acid); CDNB, 1-chloro-2,4-dinitrobenzene; TCA, Thiobarbituric acid; OPs, Organophosphates; AD, Alzheimer’s disease; PD, Parkinson’s disease; Q3G-RF, Quercetin-3-O-β-D-glucopyranoside-rich fraction.
